# Whole-genome sequencing of glioblastoma reveals enrichment of non-coding constraint mutations in known and novel genes

**DOI:** 10.1186/s13059-020-02035-x

**Published:** 2020-06-09

**Authors:** Sharadha Sakthikumar, Ananya Roy, Lulu Haseeb, Mats E. Pettersson, Elisabeth Sundström, Voichita D. Marinescu, Kerstin Lindblad-Toh, Karin Forsberg-Nilsson

**Affiliations:** 1 Department of Medical Biochemistry and Microbiology, Science for Life Laboratory, Uppsala University, SE-751 23 Uppsala, Sweden; 2 Broad Institute, Cambridge, MA 02142 USA; 3 Department of Immunology, Genetics and Pathology, Science for Life Laboratory, Uppsala University, SE-751 85 Uppsala, Sweden

**Keywords:** Glioblastoma, Cancer, Non-coding constraint, Gene regulation

## Abstract

**Background:**

Glioblastoma (GBM) has one of the worst 5-year survival rates of all cancers. While genomic studies of the disease have been performed, alterations in the non-coding regulatory regions of GBM have largely remained unexplored. We apply whole-genome sequencing (WGS) to identify non-coding mutations, with regulatory potential in GBM, under the hypothesis that regions of evolutionary constraint are likely to be functional, and somatic mutations are likely more damaging than in unconstrained regions.

**Results:**

We validate our GBM cohort, finding similar copy number aberrations and mutated genes based on coding mutations as previous studies. Performing analysis on non-coding constraint mutations and their position relative to nearby genes, we find a significant enrichment of non-coding constraint mutations in the neighborhood of 78 genes that have previously been implicated in GBM. Among them, *SEMA3C* and *DYNC1I1* show the highest frequencies of alterations, with multiple mutations overlapping transcription factor binding sites. We find that a non-coding constraint mutation in the *SEMA3C* promoter reduces the DNA binding capacity of the region. We also identify 1776 other genes enriched for non-coding constraint mutations with likely regulatory potential, providing additional candidate GBM genes. The mutations in the top four genes, *DLX5*, *DLX6*, *FOXA1*, and *ISL1*, are distributed over promoters, UTRs, and multiple transcription factor binding sites.

**Conclusions:**

These results suggest that non-coding constraint mutations could play an essential role in GBM, underscoring the need to connect non-coding genomic variation to biological function and disease pathology.

## Background

Glioblastoma (GBM) is an extremely aggressive brain tumor, characterized by high inter- and intra-patient heterogeneity [1, 2]. Despite maximal safe resection, followed by radiotherapy and chemotherapy with temozolomide (TMZ), the average survival is only 15 months [3]. Two forms of GBM are defined based on genetic mutations observed in the isocitrate dehydrogenase (*IDH1 and IDH2*) genes [4]. Primary GBM comprises 90% of the cases and is isocitrate dehydrogenase *IDH*-wild-type, while secondary GBM develops from lower grade glioma and carry mutations in IDH. Although these two types of GBM are histologically indistinguishable, they differ in genetic and clinical features [5]. Common genetic alterations in GBM include loss of the chromosome arm 10q, alterations in *TP53* and *RB*, amplifications of *EGFR* and *PDGFR*, and aberrations in RTK/Ras/PI3K signaling pathways, all of which are major known drivers of GBM pathology. Other frequent mutations include alterations in *NF1*, *PTEN*, and *MDM2* [6, 7]. GBMs are continually evolving and, within a single patient, could display multiple subtypes, gene profiles, transcriptome patterns, and methylation phenotypes, all features that could favor sub-clonal selection [8]. Based on extensive molecular classification of GBM by “The Cancer Genome Atlas” (TCGA), distinct molecular subtypes for IDH-wild-type GBMs were identified [7, 8].

Whole-genome sequencing (WGS) of GBM tumors has highlighted the importance of TERT promoter mutations in the development of the disease [9] and has been instrumental in improved understanding of clonal and sub-clonal evolution for GBM recurrences [10]. Because the absolute majority of mutations in cancer reside in the non-coding part of the genome [11], WGS also paves the way for further identification of mutations in regulatory elements such as promoters and enhancers. Evolutionary constraint across species is one of the chief indications of functional potential and has been studied across mammals [12] and vertebrates [13]. In addition, multiple other resources exist to assign function, including non-coding RNAs [14], enhancer predictions [15], ENCODE data of transcription factor binding sites, histone methylation, and DNA methylation [16]. Many of these changes accumulate in the genome all along the oncogenic process and are likely genetic or epigenetic adaptations that are prone to be conserved for a functional outcome [11].

We have performed WGS of 38 matched tumor tissue and corresponding blood samples from GBM patients, to identify novel somatic variants in regulatory regions in and around GBM genes. The catalog of exome mutations across our samples largely mirrors what has previously been described, thus serving to validate the cohort. In addition, we report here a range of non-coding constraint mutations (NCCM) that may have functional impact on the disease. Our data provides evidence of enrichment of NCCMs for GBM-associated genes, including *SEMA3C* and *DYNC1I1*, as well as in more than 1776 other genes, many of which have not previously been linked to GBM. We therefore surmise that a better understanding of the non-coding genome of GBM tumors will help in the elucidation of functional genetic and epigenetic alterations and consequently may unlock therapeutic opportunities for personalized treatment strategies.

## Results

### Patient cohort and whole-genome sequencing of matched tumor and normal pairs

**Fig. 1:**
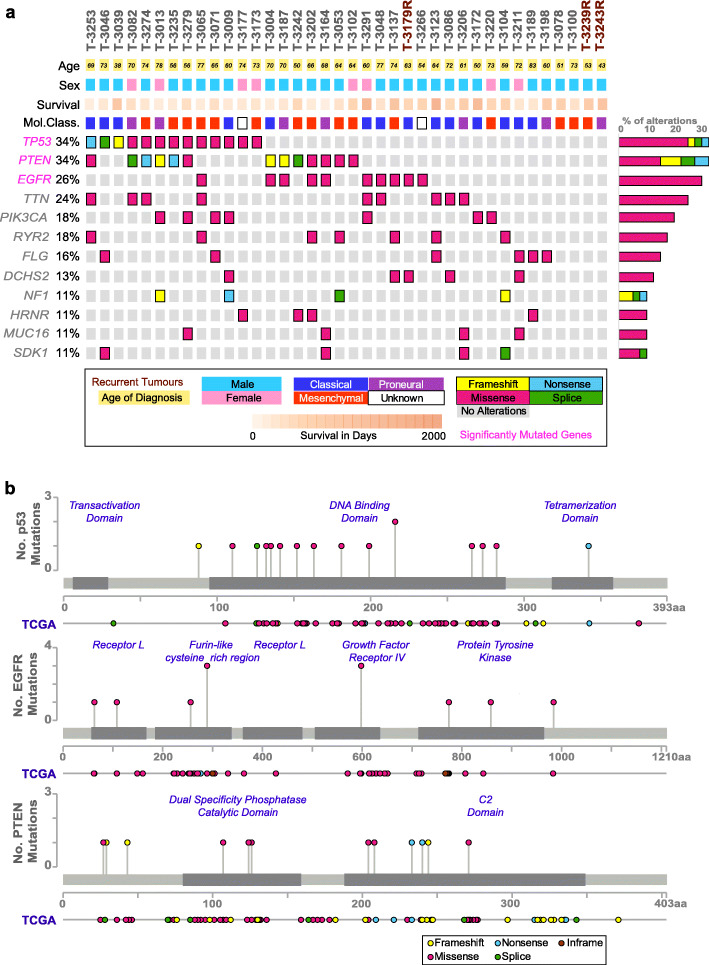
Oncoplot of the SweGBM-1 tumor cohort, and comparison of mutations in SMG/FMGs between SweGBM-1 and TCGA. **a** Per sample metadata for age, sex, and molecular type are given in the top three tracks. For the frequently mutated genes (FMGs), mutation incidence is shown as a percentage of the total cohort. Each colored brick shows the somatic alterations seen per sample in the gene (see the inset for the mutation type color code). Genes in magenta represent the significantly mutated genes (SMGs) as discerned by MuTSigCV. The right bar graph shows the rate of mutations per gene, split by mutation type. **b** For the three SMGs, non-silent mutations are observed in the same protein domains as in the TCGA-GBM dataset

The cohort designated SweGBM-1 comprised 39 IDH1^*wt*^ GBM patients: 35 patients who underwent their first surgery (treatment naïve) and four patients whose tumor had recurred. Molecular subtypes for 37 of the 39 tumors included 15 classical, 15 mesenchymal, and 7 proneural types. For two of the samples, the type could not be characterized unambiguously (Fig. 1a and Table 1). To discover somatic alterations in the SweGBM-1 cohort, matched tumor/normal pairs (*n* = 39) were sequenced using Illumina whole-genome sequencing. Alignment of the sequencing reads to the reference assembly hg19 [17] yielded depths of coverage of median 75× (range 64–89) for the tumors and 38× (range 30–66) for the matched normal.

**Table 1:** Summary of SweGBM-1 (*n* = 39) cohort with somatic point and indel mutations statistics

HGCC-ID	Glioma Grade*	Age	Sex	Survival days (diagnosis to death)	Subtype classification of the cell line	Tumor purity (aberrant cell fraction)	Depth of coverage	MuTect2-Strelka concordant SPM	MuTect2-Strelka concordant SIM
3004	IV	70	M	134	CL	89	72	7788	330
3009	IV	60	M	174	CL	72	75	9037	359
3013	IV	78	F	122	PN	83	78	8477	308
3039	IV	38	M	994	CL	98	77	7167	266
3046	IV	73	M	186	CL	71	74	8587	336
3048	IV	77	M	279	CL	99	72	9266	431
3053	IV	64	M	277	MS	64	72	4201	157
3065	IV	77	M	127	MS	84	86	14,666	450
3071	IV	65	M	309	MS	82	70	9461	386
3078	IV	51	M	716	MS	68	79	3729	170
3082	IV	70	F	314	PN	95	89	10,122	385
3086	IV	72	M	444	CL	86	72	5850	373
3100	IV	73	M	284	CL	95	68	5028	74
3102	IV	64	F	543	MS	96	75	7456	285
3104	IV	59	M	585	CL	54	73	9661	347
3123	IV	64	M	1613	CL	94	79	12,224	364
3137	IV	74	M	1088	MS	46	78	5819	227
3151^†^	IV	61	M	817	MS	73	67	230,968	1562
3164	IV	68	M	54	PN	91	81	8661	439
3172	IV	50	M	1352	CL	78	78	4312	164
3173	IV	73	F	229	MS	51	64	5345	211
3177	IV	74	F	179	n/a	99	78	10,585	522
3179^†^	IV	63	M	474	CL	64	69	4696	325
3187	IV	70	M	193	PN	62	73	4481	194
3189	IV	83	M	218	CL	98	75	7710	444
3198	IV	60	M	729	PN	75	78	7968	308
3202	IV	66	M	137	MS	84	67	5361	324
3206	IV	61	M	767	PN	71	83	4025	246
3211	IV	72	F	206	CL	61	75	5151	265
3220	IV	73	F	492	MS	70	79	4447	268
3233^†^	IV	53	M	809	MS	100	68	1590	189
3235	IV	56	M	141	MS	94	71	4570	203
3242	IV	50	M	449	MS	91	85	4336	246
3243^†^	IV	43	M	1070	PN	41	81	828	296
3253	IV	69	M	92	CL	79	78	8079	358
3266	IV	54	M	571	n/a	85	76	4767	259
3274	IV	74	M	206	MS	68	69	6447	227
3279	IV	56	M	333	MS	41	78	6320	64
3291	IV	60	F	1527	CL	89	79	7782	327

*All of the samples in the SweGBM-1 cohort are *IDH*^*wt*^

^**†**^Recurrent tumors

### Somatic copy number alterations agree with the TCGA cohort

Using the algorithm ascatNgs [18] to identify somatic copy number alterations (SCNA) present in the genome of the tumor sample relative to the matched normal, we analyzed the highly rearranged GBM landscape and found, on average, that 600 Mbp was amplified and 770 Mbp deleted per sample. The most widespread amplifications were seen in chromosome 7, covering the *EGFR* locus, in 99% of the samples. In chromosome 10, comprising the *PTEN* locus, deletions were observed in > 90% of all samples. Overall, the SCNA patterns are highly similar to what was seen in the TCGA cohort (Additional file 1: Figure S1 a, b [19, 20];).

### Variant calling and functional annotation

With the refined alignment BAM files, somatic point mutations (SPM) and somatic indel mutations (SIM) were called using MuTect2 [21] and Strelka [22]. To ensure that we had a high-quality dataset with few false-positive calls, only variants concordant between the two tools were used for the downstream analyses. A total of 256,000 SPMs and 11,127 SIMs were obtained across 38 samples (sample 3151 was hyper-mutated with 232,530 variants and hence was excluded from the above summary). Oncotator [23] was subsequently used for translational annotation of the somatic variants into coding and non-coding variant classes. Parsing the annotations for all the samples showed that > 98% of the variants were distributed across the non-coding part of the genome (Additional file 1: Figure S2a). Variant allele fraction (VAF, the fraction of reads overlapping a genomic coordinate that supports the alternate allele), however, showed no skew in the distribution for the coding and non-coding variant class categories (Additional file 1: Figure S2b).

### Protein-modifying alterations recapitulate annotations in TCGA GBM genes

Among the coding variants, MuTSigCV [24] was used to delineate significantly mutated genes (SMGs, genes that harbor more protein-modifying variants than expected by chance and are more likely to play an active role in tumorigenesis). The algorithm identified three genes, *TP53*, *EGFR*, and *PTEN* as SMGs. Applying a frequency-based approach, we identify nine more frequently mutated genes (FMGs), with non-silent variants that were found in ≥ 4 (10%) of all samples (Fig. 1a). All 12 of the FMGs/SMGs overlap with the top 20 genes seen to be mutated in the TCGA GBM dataset (Additional file 2: Table S1 [25];). In addition, 21% of mutations in the 12 proteins, encoded by these genes, are concordant with positions previously reported to have mutations in GBM (Fig. 1b, Additional file 3: Table S2). Also, 39% of the altered positions seen in these genes, including *TP53*, *EGFR*, and *PTEN*, have not been previously seen in the TCGA GBM dataset, but have been observed in other cancers [26, 27] and/or are included in the Memorial Sloan Kettering (MSK) cancer hotspot resource [28] (Additional file 3: Table S2).

### TERT promoter mutations

Frequent alterations in the promoter of the telomerase reverse transcriptase (*TERT*) gene have been described across several cancers [29, 30] including glioma [9, 31]. In the SweGBM-1 dataset, relative to the *TERT* gene start codon, > 75% of samples are observed to have mutually exclusive mutations at − 124 bp, hg19 chr5:1,295,228 C > T (24/38 samples) and at − 146 bp (hg 19 chr5, 1,295,250 C > T (4/38 samples) (Additional file 1: Figure S3). These two mutational hotspots are also seen across a multitude of cancers [32]. In our study cohort, > 75% tumors have these mutations, which is close to the 80% observed by Heidenreich et al. [31], but less than what was seen by Korber et al. [9], wherein they note that all of their primary GBM tumors have one or the other hotspot changes. Heidenreich and colleagues [31] have also shown that for primary GBMs, there is an inverse correlation between mutations in the therapeutic/diagnostic markers of isocitrate dehydrogenase 1 (*IDH1*) and alterations in the above promoter coordinates of *TERT*. Since all of our tumor samples are IDH1^*wt*^, observation of the above TERT promoter mutations in 76% of the patients is consistent with previous results.

### Enrichment of non-coding constraint mutations in the neighborhood of key GBM genes

The majority of somatic mutations across tumor samples are found in the non-coding regions of the genome, consistent with the fact that > 98% of the genome is non-coding. Most of these alterations are likely neutral passenger events and are not expected to impact the fitness of cancer cells. Nonetheless, a fraction of these non-coding changes are associated with regulatory elements of specific genes, such as promoters, UTRs, splice signals, lncRNAs and transcription factor binding sites, enhancers, and DNA methylation regions that can be expected to have roles in tumorigenesis [33]. We, therefore, decided to investigate alterations in the regulatory sequences in the vicinity of a set of genes that have roles in GBM by surveying for mutations occurring in evolutionarily constraint sequences. Accordingly, we first selected 78 key genes, known to have frequent protein-coding changes in GBM by combining the SweGBM-1 SMG/FMG and TCGA-GBM SMG gene sets (Additional file 4: Table S3).

**Fig. 2:**
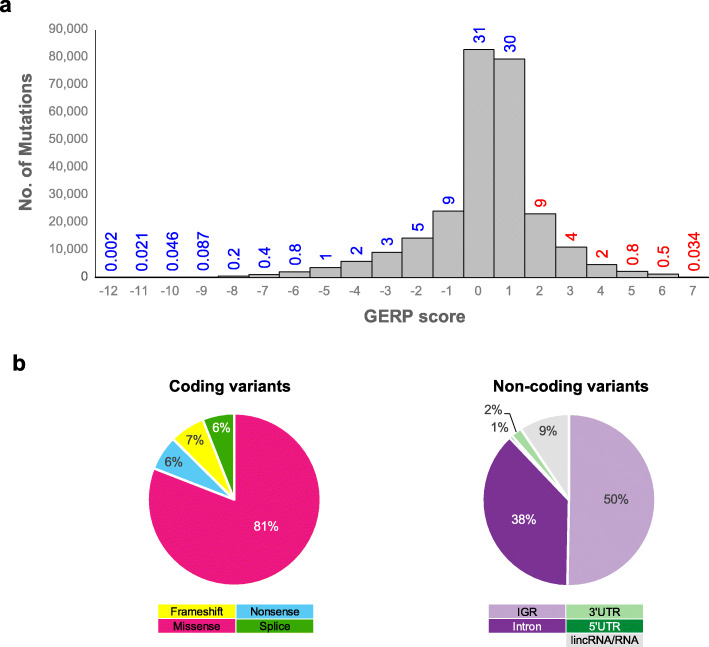
GERP score distribution for the SweGBM-1 cohort. **a** Scores range from − 12 to + 7; approximately 15% of all variants, with GERP RS ≥ 2 are deemed to be “constrained” (marked in red). **b** Distribution of variants in constrained sites by category for coding and non-coding mutations (see the inset for the mutation type color code)

Roughly 80% of genetic variants that influence gene regulation occur in cis-acting eQTLs that reside within 1 Mbp from their target genes [34–36]. Here, to be conservative, we applied a more stringent threshold and chose to examine somatic changes in intergenic regions (± 100 kbp) of the above key genes together with mutations in UTRs, introns, and non-coding RNAs. We limited this search with the 33-vertebrate constraint [13], to target variants with potential functional impact. The data thus obtained were denominated as non-coding constraint mutations (NCCMs). We used the tool GERP++ [13] for the detection of NCCM in our data set (Fig. 2a). Variants with a GERP RS value ≥ 2 were deemed to lie in constrained regions [13], and in our cohort, ~ 15% of variants (both coding and non-coding) satisfied this criterion. Figure 2b shows the distribution of variants in conserved sites, by category, for coding and non-coding regions.

**Fig. 3:**
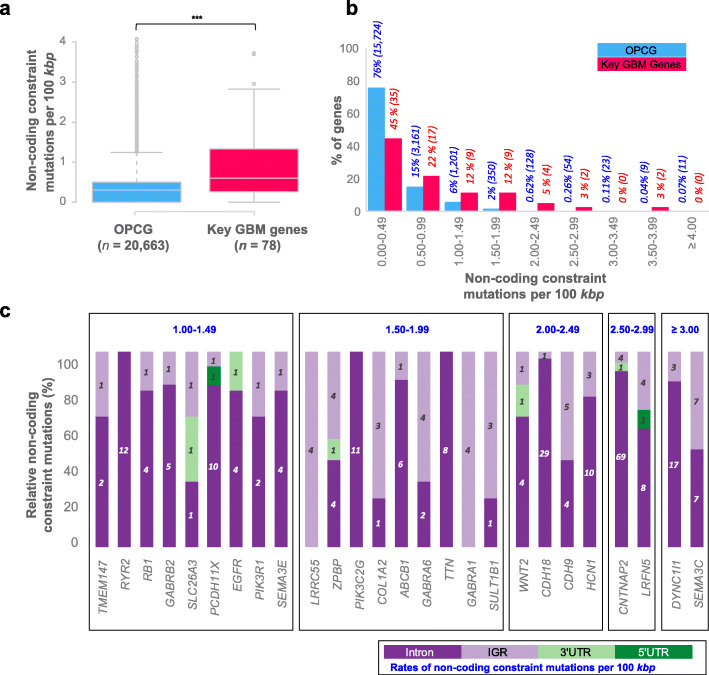
Comparison of rates of NCCMs associated with key GBM genes versus all other protein-coding genes. **a** Boxplot depicting the amount of non-coding constraint mutations per kbp for key GBM genes versus all OPCGs shows a significantly higher rate of alteration (*t* test, *P* value < 0.0001). Median, the middle data point is represented as a gray line in the middle of the boxplot and the upper whiskers represent the maximum value within 1.5 * interquartile range of the upper quartile. **b** Rates of non-coding constraint mutation for key GBM genes compared to all other protein-coding genes show the fraction of genes occurring in different rates of alteration. **c** A total of 26 key GBM genes have ≥ 1 NCCM per 100 kbp*.* See the insert for the type of mutation

We then evaluated the rate of NCCMs, normalized to the length of the queried genomic region for each of the key genes versus all other protein-coding genes (OPCG). An enrichment of NCCMs was observed in the neighborhoods of the key genes as compared to the frequency of NCCMs seen in the OPCGs (Fig. 3a, *t* test, *P* value < 0.0001). We also confirmed that the rates of non-coding constraint sites (positions that have the potential to become mutated) were uniform across the categories of key genes and OPCGs (Additional file 1: Figure S4, *t* test, *P* value = 0.09). This ensures that the enrichment we discerned is not a function of having a higher frequency of constraint sites in the flanking regions of our key genes. Of the 78 key genes, a total of 26 genes had > 1.0 NCCM per 100 kbp (Fig. 3b). Eight genes had constrained mutational frequencies that were > 2.0/100 kbp (Fig. 3c), and the four genes with the most NCMMs per 100-kbp sequence were *SEMA3C*, *DYNC1I1*, *LRFN5*, and *CNTNAP2*.

Comprehensive annotation of the NCCMs, encompassing either reported or validated regulatory elements, or both, was carried out with data from the ENCODE Analysis Hub [37] and the UCSC Genome Browser [16] (Additional file 5: Table S4). A data-driven estimate of regulatory impact per site was achieved by intersecting multiple independently curated annotation tracks (H3K4Me, H3K27Ac, DNase Clusters, Transcription Factor ChIP-seq). Given the independence between tracks, positions that re-occur in more than one track carry increased likelihood of regulatory function, giving a comprehensive overview of the regulatory DNA of the identified variants. For the key genes with ≥ 2 NCCMs per 100-kbp sequence, we observed that 85% of the NCCMs had alterations either in putative transcription factor binding sites (TFBS) (curated as well as Txn Factor ChIP validated annotations), ORegAnno elements (curated regulatory annotations), and/or other functional annotations such as DNase I hypersensitivity and/or DNA methylation in brain tissue.

**Fig. 4:**
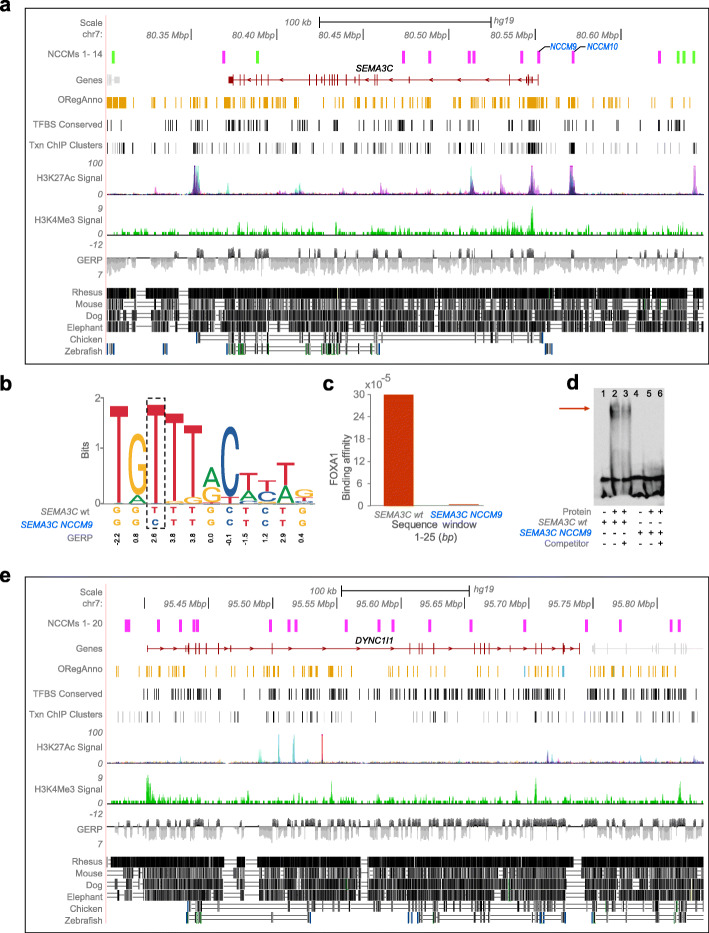
UCSC genome browser view of *SEMA3C* and *DYNC1I1*, key GBM genes with the highest rates of non-coding constraint mutations. For each gene, the NCCM track shows the mutations, color-coded by VAF scores (green VAF 1–10%; fuchsia VAF 11–45%). **a** For *SEMA3C*, the 14 NCCMs seen both in introns and in the flanking intergenic regions lie in regions of the genome that are well conserved across mammals and 13 of 14 are associated with at least one additional regulatory annotation. **b** The sequence logo (MA0148.1) of the FOXA1 TFBS shows that the SEMA3C NCCM9 mutation affects a highly conserved nucleotide that could abate the binding in the mutated site compared to the wild-type. **c** The affinity profiles, for the same mutated sequence, shows a decreased affinity for the FOXA1 transcription factor, in the mutated compared with the wild-type sequence. **d** Electrophoretic mobility shift assay of DNA protein binding for *SEMA3C* wt and *SEMA3C* NCCM9. Purified nuclear protein from GBM cell line U3065MG was tested for DNA binding to either the predicted *SEMA3C* wt region dsDNA (lanes 2–3) or the corresponding *SEMA3C* NCCM9 region dsDNA (lanes 5–6). Unlabeled dsDNA for each region was used as competitor. The total lack of shift in lanes 5–6 confirms abolition of DNA binding capacity as a consequence of the mutation. **e** In *DYNC1I1*, the majority of the 20 NCCMs are seen in the intronic regions of the gene and in regions with mammalian conservation. In addition, regulatory annotations associated with promoters and conserved TFBS are also seen

For semaphorin 3C, *SEMA3C*, a gene overexpressed in glioma and employed by glioma stem cells to promote tumorigenicity [38], we observe 14 NCCMs (in 11 patients; one or more NCCM/patient) with an equal distribution of intergenic and intronic NCCMs (7 each) (Fig. 4a, Additional file 6: Table S5). In addition, four of these NCCMs, number 9, 10, 13, and 14, were present in regions predicted to bind transcription factors (TF) and are thus potentially of high biological relevance. Since TFs recognize and bind to specific genomic DNA, we decided to investigate if any of our above regulatory mutations were likely to perturb the binding affinity using the tool “TRanscription Factor Affinity Prediction” (sTRAP module) for detecting differences in binding between two sequences [39]. NCCM9 lay in the promoter region of the *SEMA3C* gene and overlapped with a FOXA1 binding site. The sTRAP analysis revealed that the region surrounding NCCM9 is predicted to be rich in TF binding sites and that there are differences in the binding affinity between the wild-type and the mutated sequence for several TFs (e.g., ZNF354C, FOXA2, EN1, RUNX1), and with the FOXA1 factor displaying a total lack of predicted binding in the mutated sequence (Fig. 4b and c, Additional file 7: Table S6). For comparison, we performed sTRAP analysis for both the wild-type and mutated sequence for position 6 in the FOXA1 binding matrix. The position is not conserved in the matrix and did not display any differences in the binding affinities. DNA-protein binding activity for NCCM9 was further confirmed by an electrophoretic mobility shift assay (EMSA) (Supplementary Table 5, Fig. 4d). Biotin-labeled DNA probes containing the predicted DNA binding site, either for the wild-type (wt) or for the mutant sequence (NCCM9), were used, with unlabeled DNA probe as control to determine binding specificity. No binding was observed for the mutant sequence, indicating that NCCM9 leads to loss of DNA binding in this region. NCCM9 also had a VAF of 27%, indicating that it likely was an early event in the evolution of the tumor. Nine other NCCMs in *SEMA3C* also have at least one more functional annotation together with evolutionary constraint (Additional file 6: Table S5).

The *DYNC1I1* (dynein, cytoplasmic 1, intermediate chain 1) gene is known to be downregulated in glioma, and its low expression is correlated with poorer patient survival [40]. We found 20 NCCMs for *DYNC1I1* (17 intronic and three intergenic, across 16 patients, Fig. 4e, Additional file 8: Table S7). The activating epigenetic marker H3K27me3 (tri-methylation on lysine 4 of histone) is often found in promoter regions and is closely associated with transcriptionally active genes. Nine of the *DYNC1I1* variants have annotations for an H3K4me3 mark, and three of the intronic NCCMs, number 4, 10, and 15, intersected DNase I hypersensitive sites (DHSs), indicative of regions of accessible chromatin. Four NCCMs were found to lie in the transcription factor binding site (TFBS) of the HAND1-E47 heterodimer (Additional file 5: Table S4), and the 3′-UTR motif of this co-regulated gene cluster has been reported to bind to highly conserved DNA regions in lung adenocarcinomas [41]. Most of the NCCMs’ positions overlap with brain methylation signals (as discerned from the UCSF brain methylation track [42]; Additional file 8: Table S7). *DYNC1I1* NCCM15 is situated in a regulatory region that has a possible enhancer role. The sTRAP analysis for the above three NCCMs indicated significant differences between the wild-type and the mutated sequences in their binding affinity to several TFs including multiple members of the GATA family (Additional file 7: Table S6).

*LRFN5*, a fibronectin type III domain-containing protein that mediates cell-cell adhesion in a Ca^2+^ independent manner, had 14 NCCMs, all with VAF > 30% (across 13 patients, Additional file 5: Table S4). Eight of these mutations were found in introns, four in intergenic and one alteration each overlapped the 5′-UTR and lincRNA regions. NCCM5, a 5′-UTR mutation, is conserved across all 33 vertebrates and overlap with the oncogenic lncRNA CTD-2298 J14.2 [43] and TFBS for MEF2A. Three NCCMs, 5, 6 and 13, are found in large conserved regions of the genome and have TFBS predictions within 20 bp of the variant. sTRAP analysis also showed a significant shift in the binding affinity between the mutated and wild-type sequences for several TFs (Additional file 7: Table S6).

*CNTNAP2* (Contactin-associated protein-like 2), a member of the neurexin family, has been proposed as a tumor suppressor in glioma [44], and in our cohort, *CNTNAP2* showed 74 NCCMs with 29 patients having one or more variants. The majority of these NCCMs were found in introns, and several of the annotations relate to epigenetic regulation in the brain and other tissues (Additional file 5: Table S4).

### Identification of additional genes with enrichment of NCCMs

**Fig. 5:**
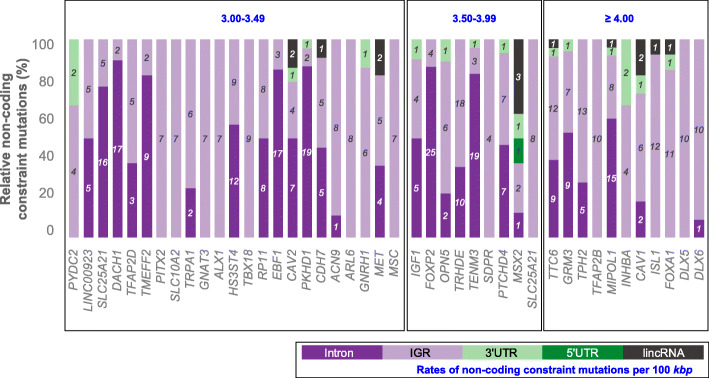
Additional genes with NCCMs enrichment. A total of 43 genes outside the key GBM gene list had ≥ 3 NCCMs per kbp (see the insert for the type of mutation)

To uncover novel genes with putative roles in GBM, we focused on the tail of the distribution of the OPCGs which showed that 1776 genes had ≥ 1.0 non-coding constraint mutations per 100-kbp sequence (Fig. 3b) and that 43 showed ≥ 3.0 non-coding constraint mutations per 100-kbp sequence. Of the 43 genes that satisfied this criterion (Fig. 5), a total of 15 have previously been reported to have altered expression patterns in GBM, while 28 genes have hitherto no known roles in the disease. Eleven of the 43 genes have ≥ 4.0 NCCMs per 100-kbp sequence (Fig. 5), and mutations for the top four genes *DLX5*, *DLX6*, *FOXA1*, and *ISL1* are distributed over promoters, UTRs, and multiple TFBS regions (Additional file 9: Table S8) and are expected to affect many transcription factor-binding motifs. We also note that approximately one third of these OPCGs with ≥ 4.0 NCCMs/100 kbp occur in clusters, one on chromosome 6 (*OPN5*, *PTCHD4*, *TFAP2D*, *TFAP2B* and *PKHD1*), two clusters on chromosome 7 (*DLX5*, *DLX6*) and (*CAV1*, *CAV2*, *MET*), and one cluster on chromosome 14 (*SLC25A21*, *MIPOL1*, *FOXA1*, *TTC6*).

**Fig. 6:**
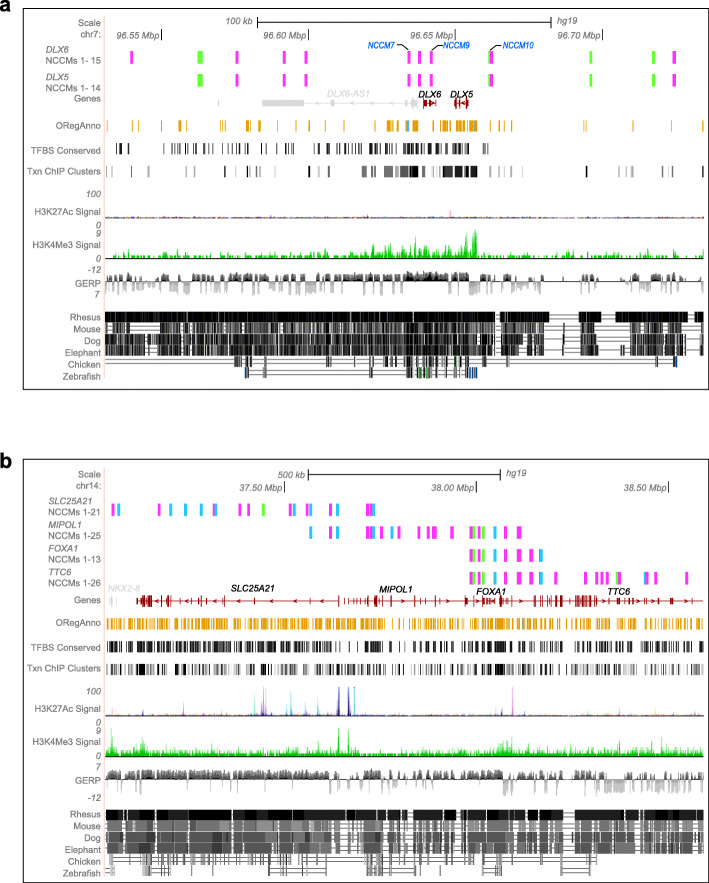
UCSC genome browser view of *DLX5* and *DLX6* and *SLC25A21*, *MIPOL1*, *FOXA1*, and *TTC6* NCCMs clusters. For each gene, the NCCM track shows mutations, color-coded by VAF scores (electric green VAF 1–10%; fuchsia pink VAF 11–45%; blue VAF > 46%). **a** Variants associated with the *DLX* genes overlap strong methylation patterns and are found in regions of mammalian conservation. **b** For *SLC25A21*, *MIPOL1*, *FOXA1*, and *TTC6*, their associated NCCMs are found in regions with regulatory potential, including promoters and TFBS

The homeobox gene *DLX5*, Distal-Less Homeobox 5, has been shown to affect glioma cell motility via the PAX6/DLX5-WNT5A axis [45]. *DLX6* is a paralog of *DLX5*, and the two genes are located in a tail-to-tail configuration on chromosome 7. Also, there is a long non-coding RNA, *DLX6-AS1*, the expression of which has been reported to correlate with worse patient outcome in GBM [46]. Together, the queried territory for *DLX5/6* showed 15 NCCMs (across 10 patients), most of which are intergenic (Fig. 6a, Additional file 10: Table S9). NCCMs 9 and 10 are located in highly conserved regions and overlap TFBS annotations for SP1 and ZIC1. The NCCMs corresponding to these positions show significant differences in their binding affinities when compared with their corresponding wild-type sequences (Additional file 7: Table S6). The position for NCCM7 is highly conserved and likely located in the promoter region of the long non-coding RNA *DLX6-AS1* (Additional file 10 Table S9).

The genes *SLC25A21*, *MIPOL1*, *FOXA1*, and *TTC6* lie in close proximity on chromosome 14 (37.1–38.6 Mbp), and therefore, the identified 56 NCCMs (across 22 patients) largely overlap between the four genes although *SLC25A21* had 14 and *TTC6* had 13 unique NCCMs, respectively (Fig. 6b, Additional file 2: Table S10). Some genes in this locus have been implicated in glioma; the *SLC25A21*gene is downregulated in GBM [47], and increased *FOXA1* transcription has been reported to promote glioma proliferation [48]. *MIPOL1*, mirror-image polydactyly 1, is vital for CNS development and is also known to have a tumor suppressor role in nasopharyngeal cancer [49]. The tetratricopeptide repeat domain 6, *TTC6*, has been shown to play a role in breast cancer, and in prostate cancer, there is evidence for a TTC6-MIPOL1 fusion [50]. The annotations associated with the NCCMs that span the four genes reveal that most have good conservation as well as multiple TFBS annotations (Additional file 11: Table S10). Here again, sTRAP analysis for the mutated sequences versus the wild-type showed significant differences in their binding affinity (Additional file 7: Table S6).

### Identification of mutational signatures

Different mutational processes leave distinctive “stamps” of somatic alterations in cancer cells; to discern these patterns, the tool “MUTation AnaLyIS toolKit” (Mutalisk [51]) was utilized. Two signatures, COSMIC 1, the so-called aging signature, and COSMIC 5, are seen as the dominant profiles across our samples (Additional file 1: Figure S5a) which is also in accord with previously detected signatures in GBM [52]. While present in many cancers, there is no clear known etiology for COSMIC 5. In addition to COSMIC1 and COSMIC5, COSMIC 11 has been reported in patients treated with the alkylating agent TMZ [52]. COSMIC11 was the overriding signature (86%), in our hyper-mutated recurrent tumor sample T-3151 (Additional file 1: Figure S5a). T-3151 is also the only sample to show a robust signal (14%) for COSMIC 14, a signature that is known to generate a very high number of somatic alterations [52]. Among the three other recurrent tumors in the cohort (T-3179, T-3233, and T-3243), which were also treated with TMZ, none exhibit COSMIC 14, and in T-3179, a minor contribution of COSMIC 11 was observed.

Interestingly, when the variants were split into coding and non-coding alterations, the former showed predominance for the aging signature, whereas the non-coding variants showed near equal distribution between the COSMIC 1 and COSMIC 5 signatures (Additional file 1: Figure S5b). Furthermore, when the mutations were divided based on being either non-constraint or constraint, we observed a significant difference in the signature distribution between the two categories (Additional file 1: Figure S5c), with COSMIC 5 dominating in the constraint changes. This dominance is also seen when the constraint dataset is parsed into non-coding changes (Additional file 1: Figure S5d).

## Discussion

Alterations in the non-coding regulatory regions of GBM largely remain unexplored. The principal goal for this study was to characterize, in a genome-wide context, the non-coding mutations that arise in GBM tumors. Here, we provide novel insights into the somatic changes affecting the regulatory landscape of GBM. Despite a relatively small cohort, our 12 significantly mutated genes (SMGs) and frequently mutated genes (FMGs) were all found among the top 20 most frequently mutated protein-coding genes in the TCGA GBM cohort. Similarly, in comparison with the same dataset, > 95% of the amplified and deleted genes are also found to be altered in our cohort. Thus, being able to mirror the SCNA landscape and the chief protein-coding mutations, as well as the mutational signatures previously found in the large TCGA cohort, lend credence to our cohort and the findings derived from it.

Mutations within the functional, non-coding regions of the genome can alter gene expression, splicing, expression of non-coding transcripts, and the epigenetic state [33]. As of today, no studies have focused on the non-coding constraint regulatory elements of the GBM genome, and thus, the impact of non-coding mutations remains largely uncharacterized. The main challenge has been to distinguish between passenger and driver mutations in the non-coding regions, where 5–10% [53] of bases have been estimated to be functional. Evolutionary conservation provides an empirical way of identifying which specific positions are important for genome function [54]. We compared 78 “key GBM genes,” identified based on frequent coding mutations, with all other protein-coding genes in the genome. Examining the sequence within and around each gene (± 100 kbp), and using a cut-off of GERP > 2 as a definition of a constrained site, we identified a highly significant enrichment of NCCMs in the neighborhood of the 78 key GBM genes with 26 genes being enriched for NCCMs (> 1 NCCMs/100 kbp). The key genes with the highest frequency of NCCMs were *SEMA3C*, *DYNC1I1*, *CNTNAP2*, and *LRFN5*.

While the NCCM frequencies in key GBM genes were skewed to the right, compared to all other protein-coding genes, the latter category still contained genes with high NCCM frequency. In total, 1776 genes had a frequency of > 1 NCCMs/100 kbp, and we further studied the 43 genes with a frequency of > 3 NCCMs/100 kbp. Overall, the distribution of NCCMs to their location category varied, with some genes (typically large ones) showing mostly intronic NCCMs, while others contained mostly intergenic variants. In an attempt to assign candidate functions to the NCCMs, we used publicly available sources of genome annotations, in addition to the evolutionary constraint, and found that most NCCMs had functional annotations, suggesting that they may indeed be driver mutations. We also noted that 91% of NCCMs in the top genes (26 key GBM genes and 43 from all other protein-coding genes) had a VAF of ≥ 10%, again supporting their ability to affect the tumor initiation and/or progression.

When assessing the potential biological importance of the analyzed variants, *SEMA3C* stood out, jointly due to its large number of NCCMs and the existing literature suggesting that overexpression of *SEMA3C* is linked to poor prognosis in several cancer types including prostate cancer [55] and GBM [56]. In the topological associated domain (TAD; chr7:80.4–81.1 Mbp) that contains the *SEMA3C* gene, we found 14 NCCMs. The *SEMA3C* gene has been shown to be regulated by several TFs, including FOXA1, GATA2, and GATA6, as well as by the androgen receptor. From our sTRAP analysis, NCCM9, in particular, clearly showed that the mutation would disrupt FOXA1 binding. This was experimentally validated in an electrophoretic mobility shift assay where the DNA-protein binding was reduced for the NCCM9 template. The DNA binding to the wild-type allele could not be competed out by an excess of unlabeled probe with the same sequence. This is a known issue for some DNA binding factors [57] but can potentially be overcome by using Multiplexed Competitor Electrophoretic Mobility Shift Assay (MC-EMSA) where the DNA binding to one probe is competed with a cocktail of probes with slightly different sequences [57]. Intriguingly, we also note that 50% of females have a *SEMA3C* mutation while only 25% of males do, possibly suggesting that the relative lack of the androgen receptor in females means that to obtain the same effect, the mutations need to occur directly in the *SEMA3C* gene to cause overexpression.

Additionally, multiple variants occur in binding sites for the GATA family of transcription factors. Given that GATA factors are known to coordinate cell survival, cellular maturation, and proliferation arrest [58], this family of genes has been anticipated to have a role in human cancers [59]. Variants for *SEMA3C*, *DYNC1I1*, and *CDH18* lie in conserved TFBS of the GATA family. GATA2 has been directly implicated in promotion of glioma through the EGFR/ERK/Elk-1 pathway [60], further indicating its potential to forward tumor development in GBM.

The discovery of non-coding driver mutations in GBM is still an emerging field and has the potential to become an integral part of clinical studies and precision medicine in the future. Here, we suggest that evolutionary constraint, combined with other genomic annotation information, could provide a useful approach to identify novel candidate GBM mutations. We hope that the findings from this study will provide a solid foundation for functional validation of novel non-coding, evolutionarily constrained candidate mutations in GBM.

## Conclusions

The results presented herein suggest that non-coding constraint mutations could play an essential role in GBM, underscoring the need to connect non-coding genomic variation to biological function and disease pathology.

## Materials and methods

### Patient cohort and ethical consent

The GBM patients for the study were included in the “Uppsala-Umeå Comprehensive Cancer Consortium” (U-CAN) biobank (www.u-can.uu.se). U-CAN is a resource for longitudinal sampling of tumor tissues, blood, and associated clinical data from cancer patients, all of which are collected with their informed consent [61]. The study was approved by the Ethical Review Board of Uppsala, Sweden (Dnr 2007/353 and addenda 2013-10-28, 2016-12-29) (Uppsala Biobank no: 827-2014-087, U-CAN: 2014-004), and all work involving human tissue samples were conducted in accordance with the Declaration of Helsinki. Matched tumor-normal specimens for sequencing were selected based on the histopathological annotation of the tumor tissues by a neuropathologist. Samples assessed as having at least 40% tumor cells were selected for WGS. The cohort was designated as SweGBM-1, and the individual samples were labeled according to their Human Glioma Cell Culture identity (HGCC [62]) with “T” and “N” prefixes used to denote tumor tissue and normal blood sample respectively. Based on specimen availability that satisfied the quality thresholds for the histopathological criterion stated above, samples from 31 male and 8 female patients, with age of diagnosis ranging from 38 to 83 years (median 65 years), were selected for the study resulting in a cohort size of *n* = 39 (Fig. 1).

### DNA preparation, library construction, and WGS

Tumor sections (3–18 sections, contingent on the extent of necrosis) of 10 μM were used to prepare tumor DNA using the “AllPrep DNA/RNA/miRNA Universal Kit” (Cat. no 80224, Qiagen, Hilden, Germany) in line with the manufacturer’s protocol. Blood DNA was using the Blood DNA kit (Cat. No 51104, Qiagen, Hilden, Germany). Tumor and normal DNA was then submitted to NGI Uppsala SNP/SEQ facility at SciLifeLab. All samples that passed the threshold values for library preparation were sequenced using the Illumina HiSeqX and True Seq PCR-free methods. Minimum target depths of coverage of 60× for the tumor and 30× for the normal were set for the sequencing, and the above protocol yielded paired-end reads of 150-bp read length for each of the matched normal and tumors in the cohort.

### Sequence data alignment

To align the raw WGS reads to the reference assembly hg19, BWA v0.7.15 [63] with default options was used. The resulting binary alignment map (BAM) files were then refined following Genome Analysis Toolkit’s (GATK [64]) recommended best practices available through the website https://software.broadinstitute.org/gatk/best-practices/. These included removal of optical duplicates with Picard’s (http://broadinstitute.github.io/picard/) utility MarkDuplicates, correction of local realignment around indels via GATK’s Indel-Realigner module, and recalibrating and reducing machine-read error induced noise from individual base quality score with GATK’s base quality score recalibration (BQSR) module (Additional file 1: Figure S6).

### TCGA dataset used for comparative analysis

The TCGA dataset, “Glioblastoma Multiforme (TCGA, Provisional),” downloaded from cBioPortal was used for all comparative analyses.

### Somatic Copy Number Alteration (SCNA) calling and comparison with TCGA SCNA data

Somatic copy number aberrations present in tumor samples were determined with ascatNGS (Additional file 1: Figure S6 [18]). For a given sample, matched refined T- and N- BAM files were provided as input data to the tool and executed with default options. Resulting “seg” output files were then used for comparison with the TCGA SCNA dataset. To assess if the SweGBM-1 copy number profiles had alterations that matched with the TCGA, GISTIC defined recurrent SCNAs, data for the latter was first downloaded from the cBioPortal, after which a custom script was run to find regions that overlapped in the two cohorts.

### Somatic variant calling and filtration

The tumor (T)- and normal (N)- BAM files were also used as input for the somatic mutation calling tools of MuTect2 v3.7-0 [21] and Strelka v1.0.15 [22]. Each of these tools output a variant calling format (VCF) file that contains somatic point and indel mutations present per matched T/N sample. To eliminate potential sequencing and germline artifacts and other sources of false-positive calls that may be present in the VCFs, a series of filtration steps were performed. Firstly, calls that were marked as germline SNPs in at least two samples in a “Panel of Normals” (PoN) dataset were filtered out. The PoN dataset was built from filtered SNPs/germline variants called by the GATK’s HaplotypeCaller across the normal samples of the SweGBM-1 cohort. Another round of filtering involved removal of germline calls that were present in the publicly available databases of dbSNP v150 [65] and SweGen Variant Frequency Dataset [66]. Since there is a possibility that in these two databases some somatic variants are erroneously marked as germline variants, calls present in either resources which also overlapped with somatic variants present in the COSMIC database [26] were “whitelisted,” i.e., not filtered out. To further minimize the rate of false-positive calls, the list of filtered calls generated with MuTect2 and Strelka was intersected. Only the somatic point mutations (SPM) and somatic indel mutations (SIM) that were in consensus between the callers were used as the final call set for all downstream analysis.

### Classification of SweGBM-1 SPM and SIM calls and identification of significantly mutated genes

To functionally classify variants into coding and non-coding categories, the SPM and SIM calls were annotated by the tool Oncotator [23]. To discover genes that may contain putative coding driver mutations, two methods were adopted: first, a statistical approach using the tool MutSigCV [24] with default options was run to get significantly mutated genes (SMGs). The counts here were then augmented with a frequency-based approach, wherein genes that were mutated in ≥ 4 tumor samples (approximately 10% of samples) were tallied to get a list of frequently mutated genes (FMGs).

### Visualization of the somatic alterations

To visualize alterations in the SMGs and FMGs, oncoprints and lollipop plots were generated using tools available on the web-based utility cBioPortal [27].

### TERT promoter mutation analysis

The TERT promoter locus encompassing known hotspot mutations on chromosome 5 at positions 1,295,228 and 1,295,250 was examined for alterations in the MuTect2-Strelka concordant dataset for all of the samples.

### Defining a key GBM gene set for the investigation of non-coding variants with regulatory potential

Alterations in key genes, especially SMGs, are known to be associated with GBM tumor initiation and/or progression. To investigate if non-coding regulatory variants of these key GBM genes might contribute to the GBM phenotype, we compiled a set of genes by pooling together the list of the SMGs (*n* = 71 [27]) from the TCGA-GBM cohort and SMGs/FMGs (*n* = 12) from the SweGBM-1, resulting in a total of *n* = 78 genes (Additional file 4: Table S3).

Non-coding variants associated with the above-described key genes were then examined in greater detail. GERP RS scores, a measure of sequence conservation, were collated for every SPM and SIM across the cohort, based on the data from the UCSC GERP RS conservation track [16]. Non-coding variants, whose position had a GERP RS score ≥ 2 and which were located within 3′- or 5′-UTRs, introns, or ± 100-kbp intergenic flanking regions, were extracted. These variants, referred to hereafter as non-coding constrained mutations, or NCCMs, were subsequently annotated with regulatory annotations downloaded from either the UCSC genome browser or ENCODE portal, or both. These include, among others, information from tracks pertaining to transcription factor binding sites (TFBS), methylation and histone modification markers (H3K4Me, H3K27Ac), regulatory markers (ORegAnno), transcription start sites, enhancer information, and chromatin immunoprecipitation assay data of DNA regions where TFs/proteins bind (Txn Factor ChIP) (Additional file 1: Figure S7).

### Statistical tests for enrichment analysis

The rate of NCCMs around key genes was compared with the same class of variants associated with all other protein-coding genes (OPCG). A *t* test was performed using the R statistical framework (R Foundation for Statistical Computing, Vienna, Austria. http://www.R-project.org/).

### Transcription factor binding affinity prediction

The sTRAP module from the TRAP tools (http://trap.molgen.mpg.de/cgi-bin/trap_form.cgi) was used to predict if regulatory sequences associated with NCCMs of key GBM genes and OPCG could alter transcription factor binding affinity to DNA. For every NCCM, 41-bp sequences were analyzed with the wild-type or the mutant allele centered. The matrices for the analysis were set to the JASPAR database, and for the background model “human promoter,” option was selected. The JASPAR database (http://jaspar.genereg.net) was used to obtain information about TF binding matrices and motifs of interest.

### EMSA (electrophoretic mobility shift assay)

For the NCCM9 and wild-type sequence in the *SEMA3C* promoter, 5′ biotin-labeled and unlabeled forward strand DNA oligos and their reverse complementary unlabeled strand (HPLC-purified, purchased from IDT (Integrated DNA Technologies) were as follows:

wt_F:GCAACAGTGGTTTGCTCTGGAGAGGAAA

wt_R:TTTCCTCTCCAGAGCAAACCACTGTTGC

NCCM9_ F:GCAACAGTGGCTTGCTCTGGAGAGGAAA

NCCM9_R:TTTCCTCTCCAGAGCAAGCCACTGTTGC

Oligos were first annealed in equimolar amounts in 1× annealing buffer (50 mM NaCl, 10 mM Tris-HCl, 10 mM MgCl2, 100 μg/ml BSA, pH 7.9 at 25 °C) in a thermo cycler by heating to 95 °C for 5 min and gradual cooling at 1 °C/min to 4 °C. Nuclear protein was extracted from patient-derived glioblastoma cells U3065MG [62] using the NucBuster™ Protein Extraction Kit (Millipore). The subsequent binding reaction of the dsDNA with nuclear protein extract was processed using LightShift™ Chemiluminescent EMSA Kit (Thermo Scientific) as per the manufacturer’s protocol and incubated on ice for 40 min for the binding reaction. Following this, 5 μl of loading buffer was mixed with each binding reaction and a total of 20 μl per reaction was then loaded per well onto a Bio-Rad Criterion gel (Bio-Rad) and electrophoresed for 90 min at 200 V. The gel was transferred to a GeneScreen Plus nylon hyrbidization transfer membrane (PerkinElmer) for 1 h at 45 V followed by UV crosslinking for 15 min with the membrane facing down on a transilluminator and additional 1 more minute with the membrane turned over. The membrane was developed using the Chemiluminescent Nucleic Acid Detection Module Kit (Thermo Scientific) and visualized on the Bio-Rad CCD camera (Bio-Rad).

### Mutational signatures discovery

The mutational signature detection for the cohort was performed with the online tool Mutalisk [51]. VCF files for the cohort were used as inputs, and the maximum likelihood with linear regression options was turned on. A specific cancer type was not selected for.

## Supplementary information

**Additional file 1: Figure S1.** Somatic Copy Number Alteration (SCNA) in SweGBM-1 matches observations in the TCGA-GBM dataset. **Figure S2.** Distribution of Coding and Non-Coding variants per sample for the SweGBM-1 cohort. **Figure S3.***TERT*^*p*^ mutational profiles for the SweGBM-1 cohort. Missense mutations at two positions in the TERT promoter observed previously in GBM datasets are seen in most samples. **Figure S4.** Boxplot of the rates of constraint bases in the internal and flanking regions of key GBM and all OPCG. **Figure S5.** Mutalisk algorithm identifies two major mutational signatures, Cosmic 1 and Cosmic 5 across samples. **Figure S6.** Workflow for variant and copy number detection in matched tumor-normal samples. **Figure S7.** Workflow for annotation of non-coding constraint mutations.

**Additional file 2: Table S1.** TCGA top 20 GBM genes that overlap with SweGBM-1 cohort.

**Additional file 3: Table S2.** Intersection of alterations in SweGBM-1 FMGs with TCGA-GBM dataset and with pan-cancer databases of COSMIC, cBioPortal and CancerHotspots.

**Additional file 4: Table S3.** List of Key GBM genes (*n* = 78), pooled from SweGBM-1 FMGs and TCGA-GBM SMGs.

**Additional file 5: Table S4.** Annotation of the NCCMs associated with the key GBM genes based on data from the ENCODE portal and the UCSC Browser databases.

**Additional file 6: Table S5.** Annotation of the NCCMs associated with the *SEMA3C* gene based on data from the ENCODE portal and the UCSC Browser databases.

**Additional file 7: Table S6.** Regulatory analysis of select Key GBM and OPCG NCCMs are shown here. The top five ranks of the most substantial difference of affinity between wildtype and mutated sequences are listed.

**Additional file 8: Table S7.** Annotation of the NCCMs associated with the *DYNC1I1* gene based on data from the ENCODE portal and the UCSC Browser databases.

**Additional file 9: Table S8.** Annotation of select genes outside the Key GBM genes’, associated NCCMs with data from the ENCODE portal and UCSC browser databases.

**Additional file 10: Table S9.** Annotation of the NCCMs associated with the *DLX5* and *DLX6* genes based on data from the ENCODE portal and the UCSC Browser databases.

**Additional file 11: Table S10.** Annotation of the NCCMs associated with the *SLC25A21*, *MIPOL1*, *FOXA1*, and *TTC6* genes based on data from the ENCODE portal and the UCSC Browser databases.

**Additional file 12.** Review history.

## Data Availability

Somatic variation sequence data has been deposited at the European Genome-phenome Archive (EGA, https://www.ega-archive.org), which is hosted by the EBI and the CRG, under accession number EGAS00001004379 [67]. Additional processed data is available at 10.17044/nbis/g000010 together with information on how to submit data access requests. Tumor tissue samples may be requested from the U-CAN biobank (https://www.u-can.uu.se).
