# Supplementary material for: Whole-genome sequencing of glioblastoma reveals enrichment of non-coding constraint mutations in known and novel genes
Source: Genome Biol. 2020 Jun 9;21:127. doi: 10.1186/s13059-020-02035-x (PMC7281935; doi:10.1186/s13059-020-02035-x)
Supplement: Supplementary file 1 — Additional file 1: Figure S1. Somatic Copy Number Alteration (SCNA) in SweGBM-1 matches observations in the TCGA-GBM dataset. Figure S2. Distribution of Coding and Non-Coding variants per sample for the SweGBM-1 cohort. Figure S3.TERTp mutational profiles for the SweGBM-1 cohort. Missense mutations at two positions in the TERT promoter observed previously in GBM datasets are seen in most samples. Figure S4. Boxplot of the rates of constraint bases in the internal and flanking regions of key GBM and all OPCG. Figure S5. Mutalisk algorithm identifies two major mutational signatures, Cosmic 1 and Cosmic 5 across samples. Figure S6. Workflow for variant and copy number detection in matched tumor-normal samples. Figure S7. Workflow for annotation of non-coding constraint mutations. [file 13059_2020_2035_MOESM1_ESM.docx]

**Supplemental Information for:**

**Whole genome sequencing of glioblastoma reveals enrichment of non-coding constraint mutations in known and novel genes**

Sakthikumar, S^1, 2*^, Roy, A^3*^, Haseeb, L^3*^, Pettersson, ME^1^, Sundström, E^1^., Marinescu, VD^1^, Lindblad-Toh, K^1, 2^, and Forsberg-Nilsson, K^3^**^†^**

**†***Corresponding author address: karin.nilsson@igp.uu.se*

**This file includes:**

**Supplemental Figures S1-S7**

**Figure S1**


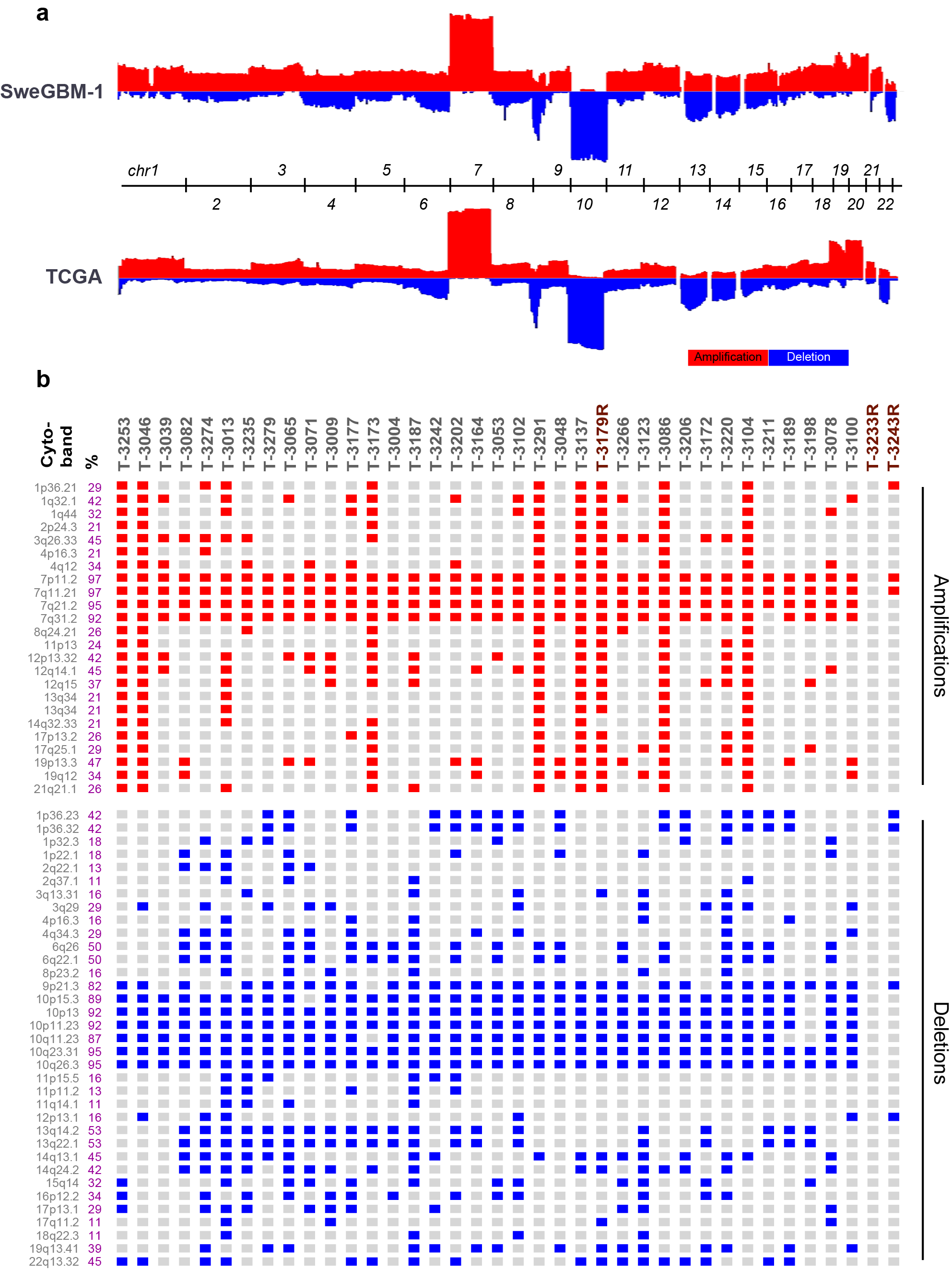


**Figure S1 – Somatic Copy Number Alteration (SCNA) in SweGBM-1 matches observations in the TCGA-GBM dataset.**

1. Integrative Genomic View of SCNAs in the SweGBM-1 and TCGA cohort shows similar patterns of amplifications and deletions across the genome.
2. Oncoplot visualization of the SCNAs seen in ≥ 10% of samples shows that several SweGBM-1 tumor samples have alterations that are seen frequently among the TCGA cohort.

**
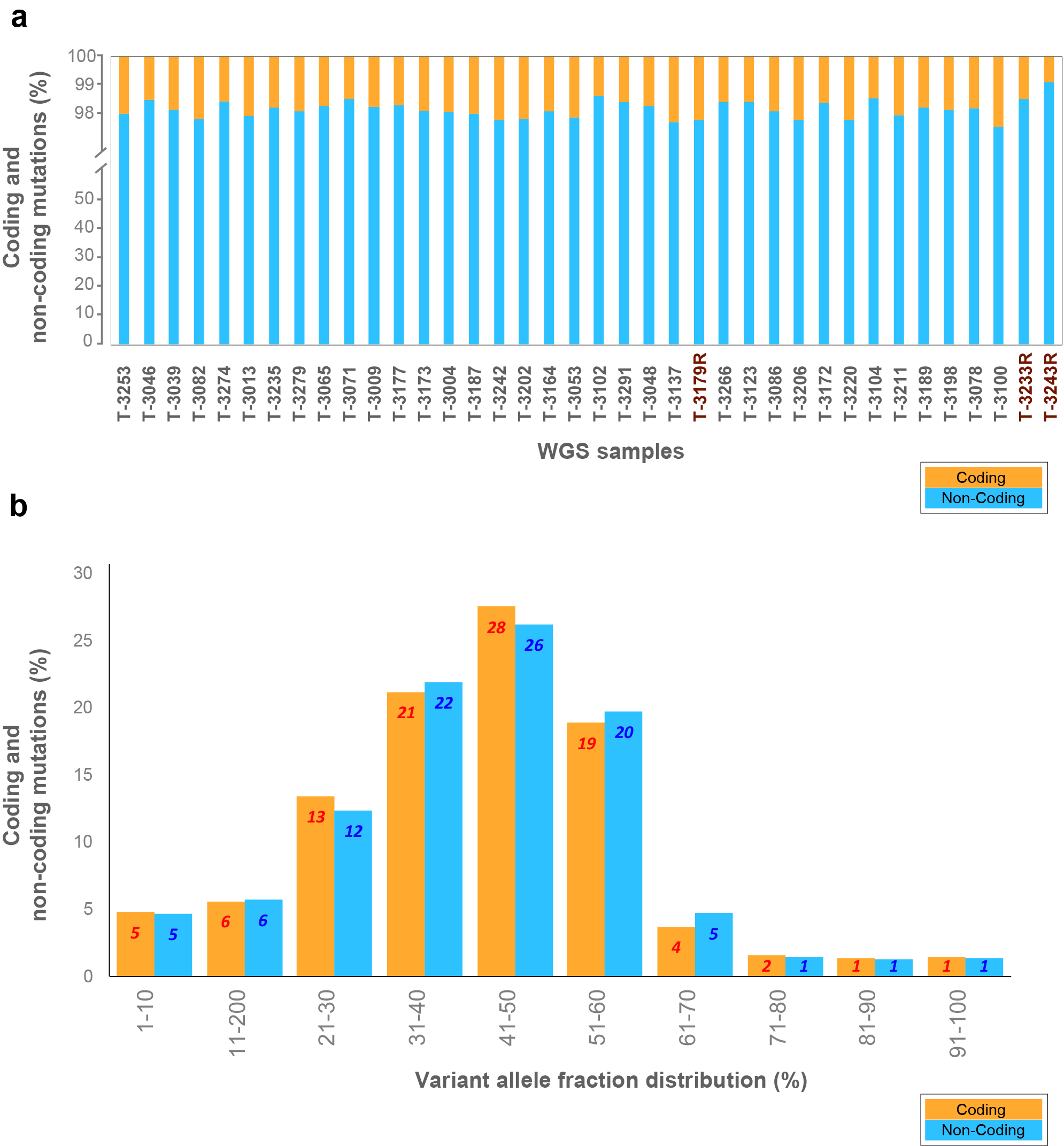
Figure S2**

**Figure S2 – Distribution of Coding and Non-Coding variants per sample for the SweGBM-1 cohort.**

1. Approximately 98% of the mutations are non-coding, in contrast to the coding variations.
2. A comparison of VAFs for coding and non-coding mutations shows no difference between these categories.

**Figure S3**


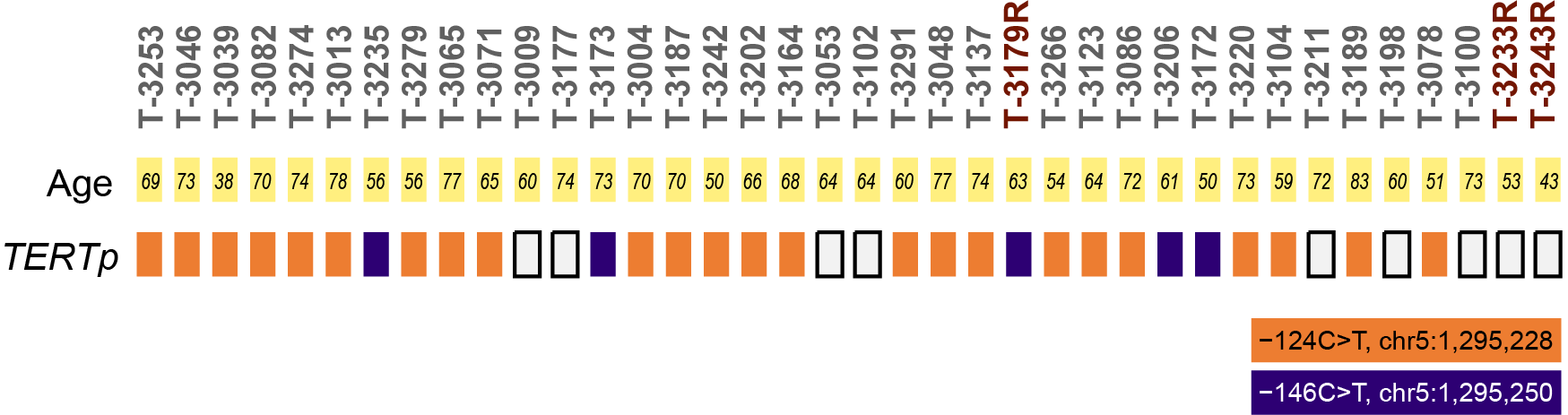


**Figure S3 –*TERT^p^* mutational profiles for the SweGBM-1 cohort.** Missense mutations at two positions in the *TERT* promoter observed previously in GBM datasets are seen in most samples.

**Figure S4**


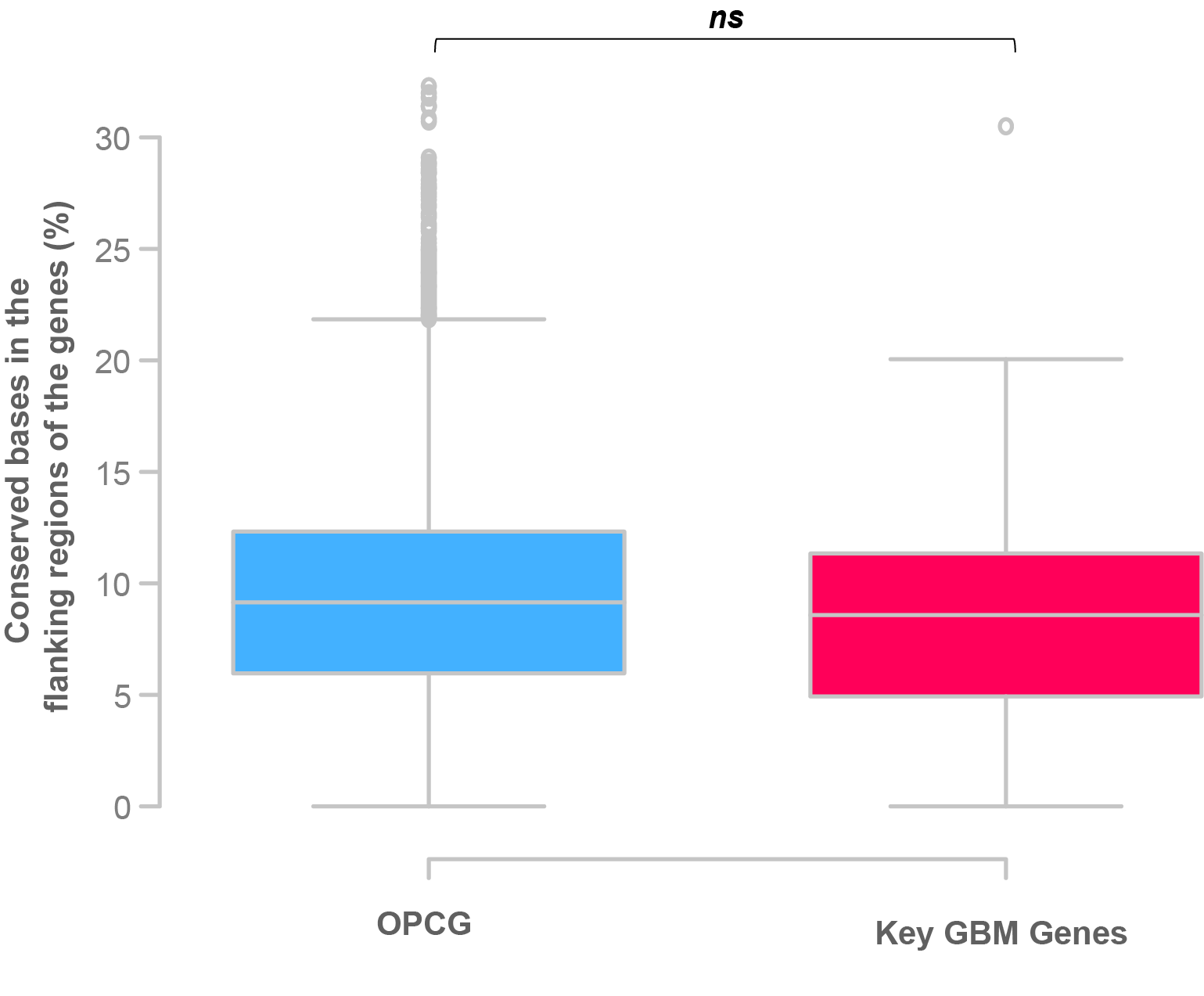


**Figure S4 – Boxplot of the rates of constraint bases in the internal and flanking regions of key GBM and all OPCG** detects no enrichment (unpaired *t-*test, *P* value > 0.5). Median, the middle data point is represented as a grey line in the middle of the boxplot and the upper whiskers represent the maximum value within 1.5 * interquartile range of the upper quartile.

**Figure S5**


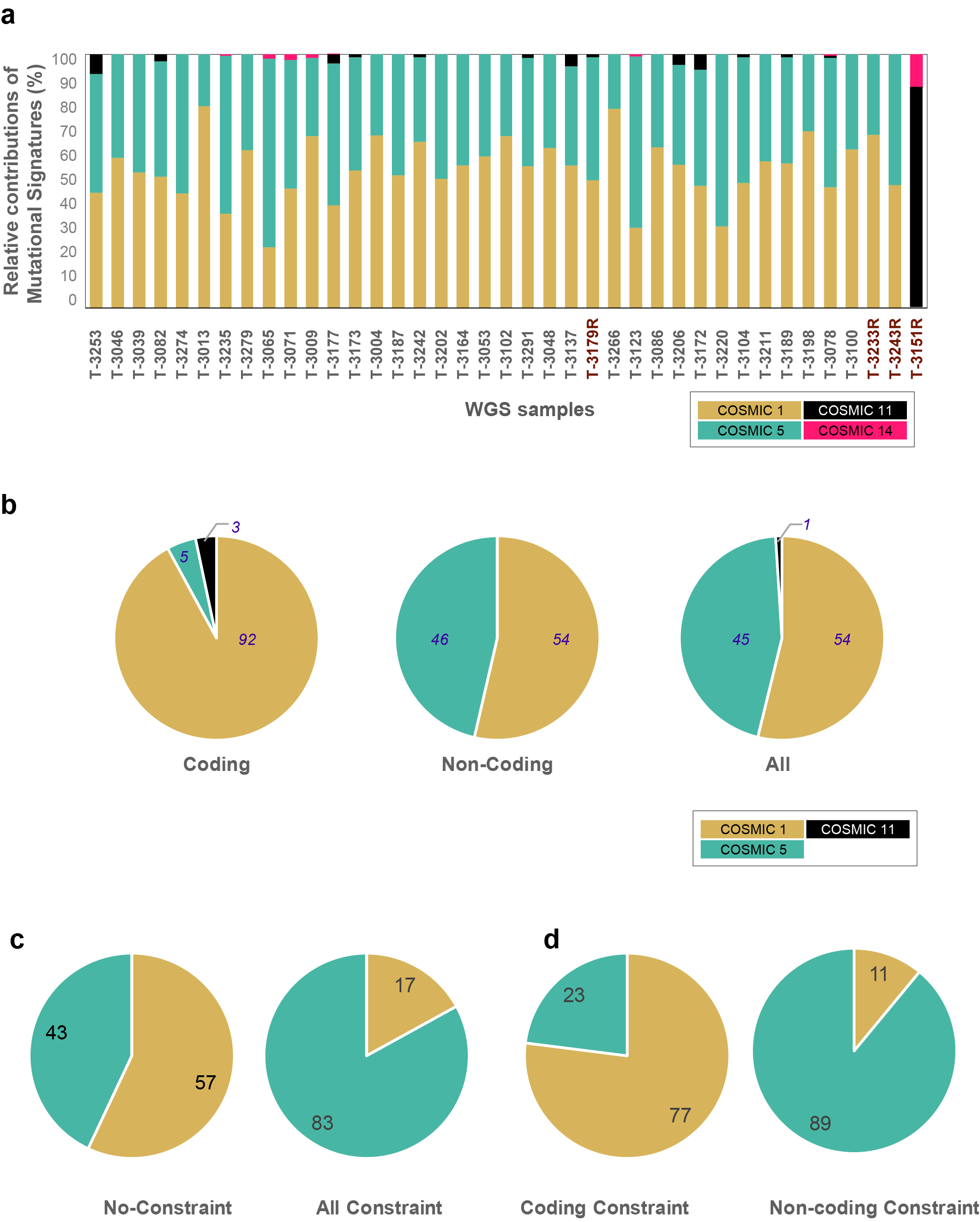


**Figure S5 – Mutalisk algorithm identifies two major mutational signatures, Cosmic 1 and Cosmic 5 across samples.**

1. Relative distribution of the Cosmic signatures, sample-wise, indicates that Cosmic 1 (*“*the aging signature”, brown), is more prevalent than Cosmic 5 (teal). For the recurrent tumor 3151 (last bar) Cosmic 11 (black) is seen as the dominant signature.
2. The pie charts show that among the coding mutations, the bulk of the changes have the aging footprint, whereas, for the non-coding mutations, there is a more even distribution between Cosmic signature 1 and 5.
3. For non-constraint mutations, there is no skew in the distribution between Cosmic signature 1 and 5, in comparison to the constraint mutations, where there is distinct bias for Cosmic5.
4. The distribution of Cosmic 1 and 5 signatures appears to be reversed between the coding constraint and non-coding constraint mutations.

**Figure S6**


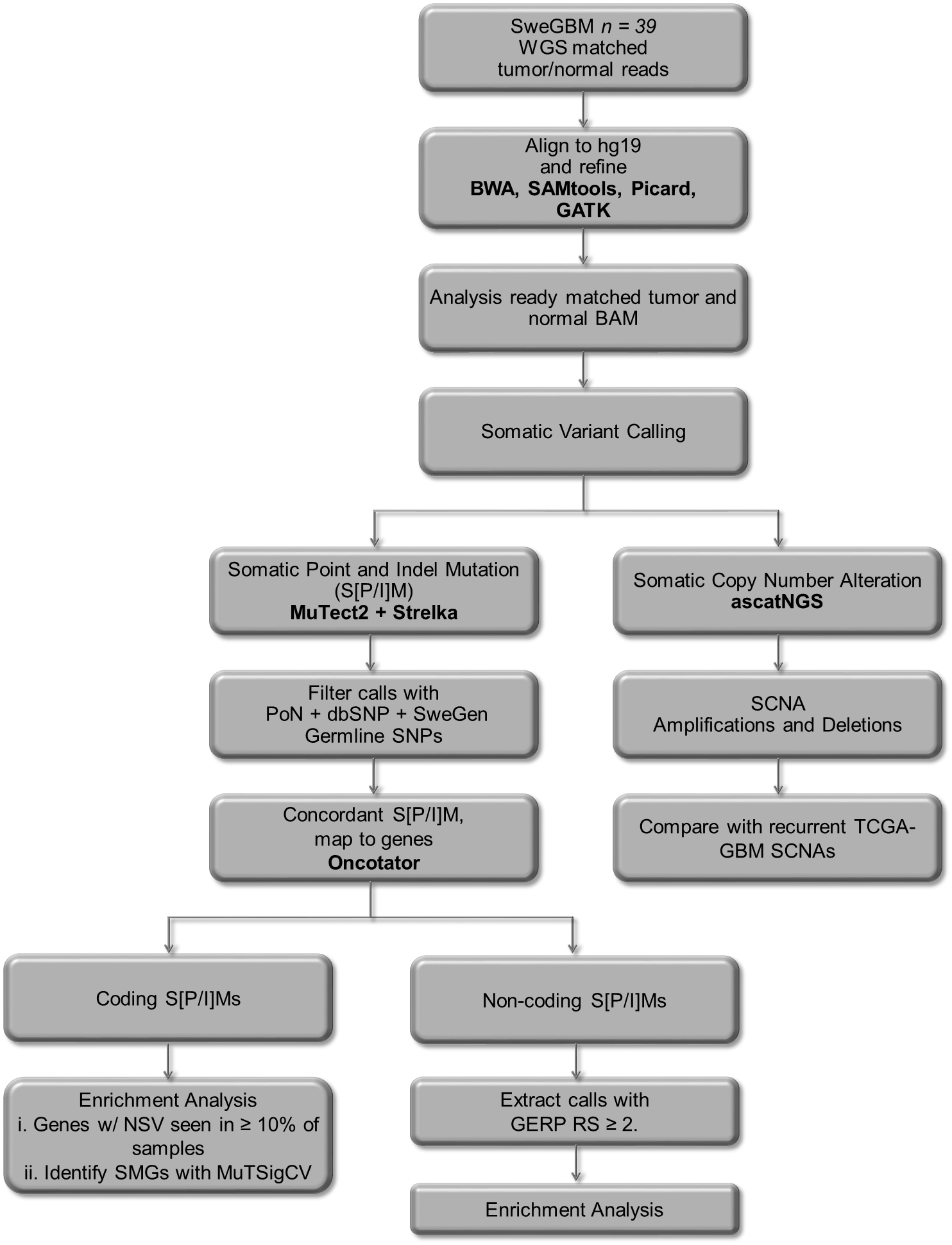


**Figure S6 – Workflow for variant and copy number detection in matched tumor-normal samples.** Sequential steps for calling SPM, SIM, and SCNA from alignment to enrichment analysis are shown here. The tools used for each step are marked in bold.

**Figure S7**


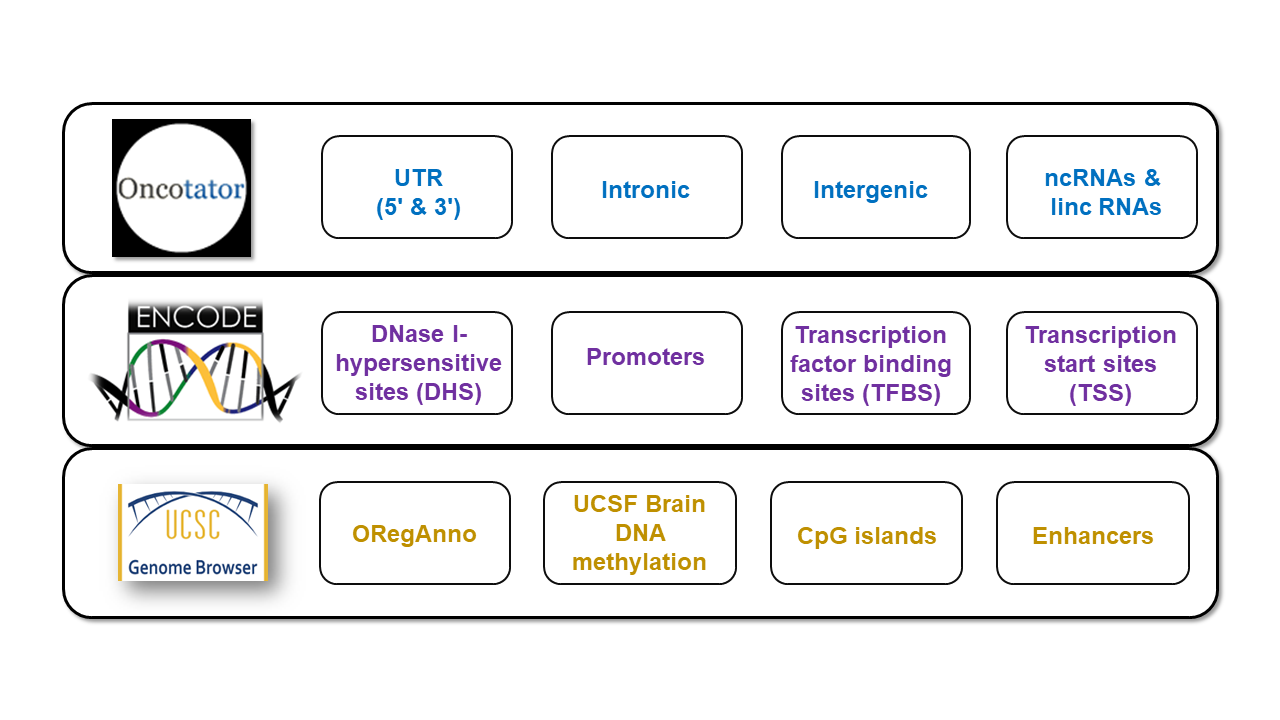
 **Figure S7 – Workflow for annotation of non-coding constraint mutations.** The NCCMs investigated were mapped with regulatory annotations obtained from the various databases listed either from the ENCODE portal or the UCSC genome browser site.
