# Supplementary material for: Whole-genome sequencing of glioblastoma reveals enrichment of non-coding constraint mutations in known and novel genes
Source: Genome Biol. 2020 Jun 9;21:127. doi: 10.1186/s13059-020-02035-x (PMC7281935; doi:10.1186/s13059-020-02035-x)
Supplement: Supplementary file 12 — Additional file 12. Review history. [file 13059_2020_2035_MOESM12_ESM.docx]

Review History

**First round of review**

**Reviewer 1**

**Are you able to assess all statistics in the manuscript, including the appropriateness of statistical tests used?**

No, I do not feel adequately qualified to assess the statistics.

**Comments to author:**

Sakthikumar et al. provide data and analysis from whole genome sequencing of a cohort of 39 IDH-wt GBM designated the SweGBM-1 cohort. Using Genomic Evolutionary Rate Profiling (GERP) and a GERP RS value >/=2 to define variants in constrained regions, they investigate the composition, frequency, and location of variants present in non-coding regions, which they designate as non-coding constraint mutations (NCCMs).  By using available data, including TCGA they demonstrate that their cohort of GBM, variant data, and copy number data are similar to those previously reported. The focus their analysis on constrained regions in non-coding regions of a select set of genes but the power of their approach may be even more impactful in the discovery of novel genes altered by NCCMs. They provide strong supporting data in support of their conclusions. Using one NCCM in SEMA3C as an example they demonstrate how the variant alters binding of the transcription factor FOXA1. Overall, the manuscript highlights the potential importance of variants in non-coding regions in GBM. The identification of constrained mutations in genes not previously identified as "key GBM" genes is a particularly important aspect of the study. The approach is potentially useful for many types of cancer.

1. The use of the term variant vs. mutation is not consistent. The term variant seems more appropriate.

2. Fig 4. The data demonstrating potential function of variant NCCM9 with respect to FOXA1 binding is powerful.  In addition to wt sequence can the authors mt a non-constrained bp to verify the specificity of the approach? Any additional functional data to support the conclusion that the NCCM variants are play a role in disease would be helpful.  

3. Fig 4 and 6.  A major emphasis of the approach is the use of GERP to identify variants of interest.  In the figures it is very difficult to assess the GERP score in relation to the variant as the distance is large.  It would be helpful to the reader to either place these closer together or provide the data in a Table. In addition, the two figures are not identical.  For example, Fig 6 contains the H3K27Ac track but Fig 4 does not. For several tracks there is no discussion or mention of how the track was used in the analysis of the variants or potential implications.

4. Fig 4. Is one of the NCCMs listed colored blue?  This is not clear as all look either green or pink.

**Reviewer 2**

**Are you able to assess all statistics in the manuscript, including the appropriateness of statistical tests used?**

Yes, and I have assessed the statistics in my report.

**Comments to author:**

In this manuscript, Sahthikumar et al employed deep whole genome sequencing to characterize the non coding variants in 38 paired GBM samples-SweGBM-1 cohort. Although the patients samples are relative low, the depth of sequencing coverage at average of 75x is reasonable and their analyses have validated the previously discovered significantly mutated genes by TCGA. In addition, TERT promoter mutations were identified in the SweGBM-1 cohort in 76% cases, which is a bit lower but consistent with previous publications. With this validation, they begin to search for non-coding variants using evolution constraint as a filter to define near 78 frequently altered GBM key protein coding genes. Of these 78 key genes, they identified NonCoding Constraint Mutations (NCCMs) in 26 genes, many of these genes with NCCMs harbor alterations in putative transcription factor binding sites (TFBS), curated regulatory annotations and other functional annotations. Using similar approaches, they further defined NCCMs in 43 other protein coding genes. The NCCMs of the top 4 genes are distributed over promoters, UTRs and TFBS. Of these 43, 28 genes are not previously associated with GBM. Their analysis indicates many of these genes are likely driver mutations.
This study is very interesting given this is the first study to focus on analyzing non coding variants in the GBM genome, their approach using evolution constraint is effective in identifying previously unknown genetic variants in GBM that may play a significant role to promote tumor progression, which is exciting but waits further rigorous experimental validation.  This study is well controlled and will provide important database and novel knowledge for the GBM field.
-Given the recently published manuscript related to deep GBM WGS (Korber et al., Cancer Cell 2019), it will be interesting to further explore this dataset to validate their key finding here, although it is not absolutely required for revision.
-the TERT mutation calling in SweGBM-1 is  a bit lower than other published data, including Korber et al., any explanation will be welcome.
-33-vertebrate constraint to target variant on page 7, line 153, may need a reference.
-for the top NCMMs genes, SEMA3C, DYNC1I1, LRFN5, CNTNAP2, DLX5/6, FOXA1 and ISL1, are these mutations associated in any specific way to GBM subtypes, given this information is already available for the SweGBM-1.
-on page 8, 187 line, VAF needs to spell out first.
-on page 10, line237, TFB should be TFBS
In figure 4, for lable TXN-CHIP, there is no corresponding text description.

**Authors Response**

**Point-by-point responses to the reviewers’ comments:**

Reviewer #1: Sakthikumar et al. provide data and analysis from whole genome sequencing of a cohort of 39 IDH-wt GBM designated the SweGBM-1 cohort. Using Genomic Evolutionary Rate Profiling (GERP) and a GERP RS value >/=2 to define variants in constrained regions, they investigate the composition, frequency, and location of variants present in non-coding regions, which they designate as non-coding constraint mutations (NCCMs). By using available data, including TCGA they demonstrate that their cohort of GBM, variant data, and copy number data are similar to those previously reported. The focus their analysis on constrained regions in non-coding regions of a select set of genes but the power of their approach may be even more impactful in the discovery of novel genes altered by NCCMs. They provide strong supporting data in support of their conclusions. Using one NCCM in SEMA3C as an example they demonstrate how the variant alters binding of the transcription factor FOXA1. Overall, the manuscript highlights the potential importance of variants in non-coding regions in GBM. The identification of constrained mutations in genes not previously identified as "key GBM" genes is a particularly important aspect of the study. The approach is potentially useful for many types of cancer.

*Response: We thank the reviewer for the positive assessment of our study.*

1.The use of the term variant vs. mutation is not consistent. The term variant seems more appropriate.

*Response: We would like to use the term variant for nucleotide changes present in the germ-line, while the term mutation is reserved for nucleotide changes appearing in the tumor. We have looked through the manuscript to ensure consistency.*

2. Fig 4. The data demonstrating potential function of variant NCCM9 with respect to FOXA1 binding is powerful. In addition to wt sequence can the authors mt a non-constrained bp to verify the specificity of the approach?

*Response: We agree that the specificity of the in silico prediction of NCCM9 perturbing FOAX1 binding should be further validated. To accomplish this, we performed sTRAP not only on the observed mutation (position 3 in the matrix) but also for position 6, which has low conservation in the FOXA1 matrix. No differential predicted binding was seen the wt and in silico mutation.*

3. Any additional functional data to support the conclusion that the NCCM variants are play a role in disease would be helpful.

*Response: We agree with the reviewer that this is important, and have therefore performed functional validation of the NCCM9. The DNA-protein binding activity was confirmed by an EMSA. This is introduced as new Figure 4d. Biotin-labelled DNA probes containing the FOXA1 consensus binding sequence, either for the wild-type (wt) or the mutant sequence (NCCM9) was used, with unlabelled DNA probe as control to determine binding specificity. No shift was observed for the mutant sequence, indicating that the binding reaction is specific and that the mutation in NCCM9 leads to loss of binding for the FOXA1 transcription factor. While this does not, by itself, provide evidence that NCCM9 supports glioblastoma cell fitness, it shows that the mutation causes the predicted perturbation of gene regulation. The follow-up study of this manuscript will analyse the role for NCCM9 and other NCCMs in GBM in greater detail.*

4. Fig 4 and 6. A major emphasis of the approach is the use of GERP to identify variants of interest. In the figures it is very difficult to assess the GERP score in relation to the variant as the distance is large. It would be helpful to the reader to either place these closer together or provide the data in a Table.

*Response: We agree with the reviewer that the GERP scores for the NCCMs should be visualized more clearly. Given the density of information in the UCSC genome browser views of Fig. 4 and 6, we choose to instead introduce color-coding for GERP scores in the supplementary tables where the NCCMs are listed. (See supplementary table S5 and tables S7-10). In this way, comparisons between GERP scores and VAF can easily be made for each NCCM.*

5. In addition, the two figures are not identical. For example, Fig 6 contains the H3K27Ac track but Fig 4 does not.

*Response: We have now ensured that the same tracks are consistent between figures, also using the same scales for the axes.*

6. For several tracks there is no discussion or mention of how the track was used in the analysis of the variants or potential implications.

*Response: We have attempted to clarify this in the revised version of the manuscript, particularly in the results section and the methods section. In the Results we write “Overlaying of annotations from multiple tracks (H3K4Me, H3K27Ac, DNase Clusters, Transcription Factor ChIP-seq) that are relevant to the regulation of transcription and that complement each other was performed to compile a high confidence dataset. The data curation for these tracks are independent of each other, and multiple tracks suggest a regulatory function for a given region, giving a comprehensive overview of the regulatory DNA of the identified variants.” In our paper, we chose to highlight some of the NCCMs in genes that had the highest density of NCCMs per 100 kbp in the text, while the complete set of NCCMs with their annotations are found in the supplementary tables.*

7. Fig 4. Is one of the NCCMs listed colored blue? This is not clear as all look either green or pink.

*Response: To clarify the NCCM track, we changed the colors and now show mutations color-coded by VAF scores with green for VAF1-10% and fuchsia for VAF 11-45%.*

Reviewer #2: In this manuscript, Sahthikumar et al employed deep whole genome sequencing to characterize the non coding variants in 38 paired GBM samples-SweGBM-1 cohort. Although the patients samples are relative low, the depth of sequencing coverage at average of 75x is reasonable and their analyses have validated the previously discovered significantly mutated genes by TCGA. In addition, TERT promoter mutations were identified in the SweGBM-1 cohort in 76% cases, which is a bit lower but consistent with previous publications. With this validation, they begin to search for non-coding variants using evolution constraint as a filter to define near 78 frequently altered GBM key protein coding genes. Of these 78 key genes, they identified NonCoding Constraint Mutations (NCCMs) in 26 genes, many of these genes with NCCMs harbor alterations in putative transcription factor binding sites (TFBS), curated regulatory annotations and other functional annotations. Using similar approaches, they further defined NCCMs in 43 other protein coding genes. The NCCMs of the top 4 genes are distributed over promoters, UTRs and TFBS. Of these 43, 28 genes are not previously associated with GBM. Their analysis indicates many of these genes are likely driver mutations. This study is very interesting given this is the first study to focus on analyzing non coding variants in the GBM genome, their approach using evolution constraint is effective in identifying previously unknown genetic variants in GBM that may play a significant role to promote tumor progression, which is exciting but waits further rigorous experimental validation. This study is well controlled and will provide important database and novel knowledge for the GBM field.

*Response: We thank the reviewer for the positive comments about our study.*

1.Given the recently published manuscript related to deep GBM WGS (Korber et al., Cancer Cell 2019), it will be interesting to further explore this dataset to validate their key finding here, although it is not absolutely required for revision.

*Response: We agree that the suggested exploration of additional GBM WGS data for NCCM analysis would be highly interesting. In the next phase of our project, we aim to expand our analysis to the GBM WGS data in TCGA, are in the process to ask Korber et al to access their data as well.*

2. the TERT mutation calling in SweGBM-1 is a bit lower than other published data, including Korber et al., any explanation will be welcome.

*Response: In our study cohort, > 75% tumors having these mutations is close to the 80% observed by Heidenreich et al., though less than what was seen by Korber et al., wherein they note that all of their primary GBM tumors have one or the other hotspot changes. Presumably, the limited sizes of our cohort, 39 GBM patients, and that of Korber et al, 21 GBM pairs, may account for some difference between our percentages. The Heidenrich cohort consisted of 165 GBM.*

3. 33-vertebrate constraint to target variant on page 7, line 153, may need a reference.

*Response: We have added the correct reference: Davydov EV, Goode DL, Sirota M, Cooper GM, Sidow A, Batzoglou S: Identifying a high fraction of the human genome to be under selective constraint using GERP++. PLoS Comput Biol 2010, 6:e1001025.*

4. for the top NCMMs genes, SEMA3C, DYNC1I1, LRFN5, CNTNAP2, DLX5/6, FOXA1 and ISL1, are these mutations associated in any specific way to GBM subtypes, given this information is already available for the SweGBM-1.

*Response: This has now been addressed, but we found no association between molecular subtypes and the NCCMs in the genes on top of the list, in our cohort. To enable the reader to easily assess whether a patient’s GBM is proneural, classical or mesenchymal, we have added a column with this information in the supplementary tables listing the NCCMs for each gene/cluster of genes.*

5. on page 8, 187 line, VAF needs to spell out first.

*Response: This has been addressed.*

6. on page 10, line237, TFB should be TFBS

*Response: This has been addressed.*

7. In figure 4, for label TXN-CHIP, there is no corresponding text description.

*Response: Due to space constraints we chose not to explain the individual tracks in the figure legend but have instead clarified this in the results and materials sections. Results: “Overlaying of annotations from multiple tracks (H3K4Me, H3K27Ac, DNase Clusters, Transcription Factor ChIP-seq) that are relevant to the regulation of transcription and that complement each other was performed to compile a high confidence dataset. The data curation for these tracks are independent of each other, and multiple tracks suggest a regulatory function for a given region, giving a comprehensive overview of the regulatory DNA of the identified variants.”. In the methods section, it reads as follows: “…annotated with regulatory annotations downloaded from either the UCSC genome browser or ENCODE portal, or both. These include, among others, information from tracks pertaining to transcription factor binding sites (TFBS), methylation and histone modification markers (H3K4Me, H3K27Ac), regulatory markers (ORegAnno), transcription start sites, enhancer information and chromatin immunoprecipitation assay data of DNA regions where TFs/proteins bind (Txn Factor ChIP).”*

**Second round of review**

**Reviewer 1**

The authors have addressed the previous comments properly.

**Reviewer 2**

the authors have satisfactorily addressed all the prior concerns
